# Duty hours as viewed through a professionalism lens

**DOI:** 10.1186/1472-6920-14-S1-S15

**Published:** 2014-12-11

**Authors:** Shiphra Ginsburg

**Affiliations:** 1 Department of Medicine and Wilson Centre for Research in Education, Faculty of Medicine, University of Toronto, Toronto, Ontario, Canada

## Abstract

Understanding medical professionalism and its evaluation is essential to ensuring that physicians graduate with the requisite knowledge and skills in this domain. It is important to consider the context in which behaviours occur, along with tensions between competing values and the individual’s approach to resolving such conflicts. However, too much emphasis on behaviours can be misleading, as they may not reflect underlying attitudes or professionalism in general. The same behaviour can be viewed and evaluated quite differently, depending on the situation. These concepts are explored and illustrated in this paper in the context of duty hour regulations. The regulation of duty hours creates many conflicts that must be resolved, and yet their resolution is often hidden, especially when compliance with or violation of regulations carries significant consequences. This article challenges attending physicians and the medical education community to reflect on what we value in our trainees and the attributions we make regarding their behaviours. To fully support our trainees’ development as professionals, we must create opportunities to teach them the valuable skills they will need to achieve balance in their lives.

[P]rofessionalism has no meaningful existence independent of the interactions that give it form and meaning. There is great folly in thinking otherwise.

Hafferty and Levinson (2008)[1]

Understanding and evaluating professionalism is essential to excellence in medical education and is mandated by organizations that oversee medical training [2]. Historically, attention has been focused largely on the professionalism of individual students or residents, at least for the purposes of evaluation. Yet there is now a growing appreciation that professionalism can be defined, understood, and evaluated from multiple perspectives [3]. Importantly, context has been recognized as critical to shaping trainees’ behaviours, and hence as important to our understanding of them [4]. A restriction in duty hours for trainees is clearly an important environmental and contextual factor to consider in evaluating professional behaviour. In this paper I will review some key issues with respect to understanding and evaluating professionalism, and then discuss these in the context of duty hour reform. Readers should note that this is not intended to be a comprehensive review of the literature of either professionalism or duty hour reform, but rather a critical narrative review that uses selected articles.

## Understanding and evaluating professionalism

Identifying unprofessional conduct in medical students and residents is as important as identifying deficiencies in other domains of their knowledge or skill. Some research suggests that the occurrence of unprofessional behaviour during medical school and residency might be predictive of disciplinary actions during a physician’s career in practice [5,6], and at least one study has shown that it is possible to improve behaviours with targeted interventions [7]. That said, professionalism has proved to be more difficult to evaluate than other domains, and red flags that are noted during training account for only a small proportion of attributable risk [8].

In 2000 my colleagues and I published a review paper that attempted to reconceptualize the evaluation of professionalism [9]. After a comprehensive literature review, we argued that the problem was the evaluation methods themselves, in that they did not focus sufficiently on context, values conflicts, or the resolution of conflicts, and thus were unable to fully represent an individual’s professionalism. I will outline these issues briefly and then discuss how they are relevant to the discourse surrounding duty hour reform.

First, we argued that evaluations should focus on behaviours rather than attitudes or personality traits. The prevailing wisdom at the time was that personality traits are not good predictors of behaviour, and that behaviours were more objective in the first place: everyone can see the same things and thus readily agree on them. Context refers to the situation: Where and when did the behaviour occur? What else might have been going on at the time, internally or externally? Context has been studied extensively and is known to shape behaviours.

The next issue to consider is that of conflict between equally worthy values, such as honesty versus confidentiality, or altruism versus self-care. Which one prevails will depend on the situation, yet evaluations do not include this consideration. Finally, we focused on resolution: How does an individual approach a situation and come to a decision about how to act? We felt that if we knew more about the process of resolution we would be better able to predict future behaviour.

Although these remain strong arguments, we have come to realize that focusing solely on behaviours is perhaps misguided. A series of studies using standardized professionalism dilemmas found that how and why we consider behaviours to be professional or unprofessional is quite variable [10-13]. By way of illustration, consider the following vignette:

A medical team is thrilled that they’re done by 5 p.m, on a Friday afternoon, and they all decide to go out for drinks. The clerk [senior medical student] arrives, and they invite him along, as it’s his last day. However, he has not quite finished his work—he has a patient that he’s worried about, who has high blood sugars and may need insulin over the weekend. The resident tells the clerk that it’s no big deal, it can wait until Monday. When the student protests, the resident tells him again not to worry, that there’s an on-call team that can deal with it, and then asks if he’s coming for drinks [14].

In one study, 30 faculty attendings were asked what they thought a student should and should not do in this situation, and much disagreement was found [11]. Many options were suggested by participants, but there was no “right answer” endorsed by all. For example, 13 thought the best thing for the student to do would be to go to another resident or the person on call; an additional 12 felt that this would be acceptable; 5 did not mention this option at all. Six thought the best option would be for the student to pursue the matter with his attending staff, while another 10 thought that would be acceptable. However, 2 explicitly stated that the student should definitely not take this course of action.

In responding to this and other vignettes, faculty were found to have differing interpretations and applications of abstract principles such as honesty, and that these principles were interpreted and evaluated in different ways depending on how the attending construed the situation. For example, one attending might evaluate a student negatively for lying to a patient and not disclosing a test result, whereas another might take a favourable view of that same lie, if he or she felt that, in the given situation, it was intended to protect the patient from unnecessary anxiety in the short term [12]. Taken more broadly in the context of other work, it is apparent that the same behaviour can be viewed and evaluated entirely differently depending on the intricacies of the situation [14].

Thus, if we focus only on behaviour without taking context into account, we risk making what social psychologists refer to as the fundamental attribution error: that is, we underestimate the influence of the *situation* and greatly overestimate the influence of the *person.* Thus we inappropriately attribute a behaviour much more to what we perceive as a person’s traits than to the situation [15]. This is considered inappropriate, since many studies have shown that, although people generally act in accordance with their attitudes, these actions are highly susceptible to external influence [4,16]. When the social pressure to act in a way that is discordant with one’s values is high, and the required behaviour is difficult (a familiar situation, perhaps, in medical education), attitudes end up accounting for only a small proportion of the variance in the range of behaviours that we see. Simply put, most of the behaviours we observe are shaped by the situation.

Unfortunately, most of the time we do not take situational factors into account in evaluating professionalism. In one study, disagreement among faculty persisted even when the rationale behind students’ behaviour was made explicit [13]. Further, attendings expressed tension with respect to what was more important – the behaviour or the reasoning behind it – which added to the difficulties of evaluation. These and other studies have illustrated how difficult it is for faculty to evaluate learners’ behaviours and judge whether they are indicative of professionalism.

This situation is amplified in the context of duty hour reform. Suppose that the vignette presented above, in which a clinical clerk wants his resident to help him with a patient, takes place not on a Friday at 5 p.m., but post-call, at 10 a.m. on a Thursday, when it is the scheduled time for handover. The residents have not slept all night. What do we think is the right thing to do now? How and why might we respond differently to this variation on the scenario?

## Duty hour regulations

Changes in duty hours for residents is a new context that must be considered in understanding professionalism. The regulation of duty hours, such as by the ACGME (Accreditation Council for Graduate Medical Education), has raised concerns that professionalism would be undermined. The authors of a prominent editorial posited that limits on duty hours would “disrupt one of the ways we have taught young physicians critical values,” such as personal responsibility to one’s patients, “above and beyond work schedules and personal plans” [16]. These authors suggested that we “risk exchanging our sleep-deprived healers for a cadre of wide-awake technicians” [17]. Another article, provocatively entitled “Duty hours versus professional ethics,” stated that our duty to patients “does not end simply because a timer has gone off” and that “to hold otherwise is to ignore a fundamental precept of the profession, that physicians must put the patient’s interests above all others” [18]. Clearly, these authors were concerned about a “shift-work mentality” and a corresponding decline of professionalism. A third, somewhat more nuanced, article expressed concern that these regulations “took away [residents’] control, preventing them from making the decisions that characterize a professional ” [19]. I will return to this theme a little later.

As the ACGME limits on duty hours were rolled out in practice, several research studies examined their impact on faculty. Arora and colleagues found that, in comparison with the days before regulation, Internal Medicine attendings reported that they had fewer hours for teaching, were less satisfied with their own professional development, hours worked and level of stimulation, and felt that residents were becoming more dependent on them [20]. In another study in Internal Medicine, key clinical faculty reported a decline in residents’ professionalism post-regulation, especially in the area of accountability, but also with respect to resident–patient relationships and an ability to place patients’ needs above self-interest [21]. The issue of accountability is interesting, in that my research team also found (in two recent qualitative studies exploring the language that Internal Medicine faculty use to describe their trainees) that the most common theme that arose in attendings’ language was “work ethic” [22,23]. Positive examples included coming in early and staying late, being responsible, and taking ownership of patients; negative examples included treating work as a 9 to 5 job or “punching the clock.” This underscores the importance that we, as faculty attendings, place on the concepts of accountability and “work ethic,” and how we may inadvertently (and, potentially, detrimentally) be using these as a surrogate for professionalism in general.

In General Surgery, interesting research suggested that a “new” professionalism was expected to develop, emphasizing teamwork and shared responsibility for patients [24,25]. However, Coverdill and colleagues found that, more than five years later, residents still felt that working longer hours was more professional. In one study, only a minority thought it was acceptable to pass off work to night teams even where they existed, and few reported receiving guidance on how to make stay-or-go decisions [26]. Further, residents wanted to be able to make their own decisions – relying on their own discretion and their own take on a situation, including the availability of resources, work-load, etc. – as part of their developing professionalism. This finding parallels that of a recent study in Internal Medicine, in which a large majority of incoming residents wanted to retain the ability to make their own decisions about exceeding shift limits [27]. In that study, residents were more likely to feel that a well-rested intern who did not know the patient, versus a fatigued intern who did, should carry out tasks such as placing an arterial line or assessing shortness of breath. However, when it came to having an end-of-life discussion, their preference between the two was split.

Finally, a seminal, three-month ethnographic study of Internal Medicine and General Surgery residents (aptly titled “To leave or to lie?”) found no evidence of a shift-work mentality [28]. The authors found that residents did not blindly obey duty hours; rather, they were dictated by the nuances and intricacies of each situation. Further, there were numerous instances in which residents lied when reporting their duty hours in order to protect themselves (e.g., if they frequently stayed past shift limits, would they be seen as inefficient?). At times, pressure on residents was exacerbated by their awareness of the consequences of violating duty hour regulations, such as loss of accreditation of their program, loss of government funding, etc.

## What do we value?

This research should stimulate reflection and prompt us, as attending physicians, to question what we value and how we evaluate. Consider the hypothetical case of a resident who comes in early every day, regularly stays past 6 p.m. on non-call days, and often exceeds post-call work hour guidelines to take care of all loose ends before signing out. Could this be an example of altruism? Dedication? Commitment? These behaviours could be construed as exemplifying the values we hold most dear: accountability, ownership, and an excellent work ethic. Alternatively, they could indicate that this resident is inefficient, distracted, or poor at time management, or that he or she is neglecting self-care and is at risk of burnout. It is also possible, of course, that the resident is behaving in this way to convey an image – one that we obviously seem to value – of excellent professionalism. It is difficult to think of an appropriate response here, as this sort of behaviour is usually assumed to reflect well on residents: we thank and commend them for their hard work. On the other hand, are we then condoning and encouraging residents to continue to violate the guidelines? And what is the option then – to discourage this behaviour, potentially inhibiting residents’ dedication and commitment and, at the same time, losing critical opportunities to teach them how to make these decisions once they are out in practice?

At this point there truly may be more questions than answers. For example, is there a “new” professionalism, and if so, is it what we think it is? Is it a good thing or a bad thing – and for whom? Attending physicians should also consider how to reconcile their own perceptions and experiences with respect to what it means to “be professional” with the current reality. And, finally, how do we go about assessing our residents’ professionalism without risking misattributions in either a positive or negative direction?

## Conclusions

To return to the framework I presented at the beginning of this article, the new context is that house staff have more limited duty hours than their attendings did during their own training, and duty hours will likely become even more restricted over time. The conflicts that arise from this situation are numerous, and require a balance to be struck between or among potentially competing values such as continuity of care, independence of professional judgment, opportunities for teaching, compliance with regulations, the need for sleep, professional identity, self-image, and many more. And, critically, it is quite possible that the way in which learners resolve these conflicts will become less visible over time, especially if we enforce compliance with duty hour regulations. This might result in lost opportunities to teach residents the life-long skills they will need to balance work with their personal and other professional interests. Decisions about when to come in to work, when to go home at the end of a day, and how to manage time between inpatient and ambulatory care, or between patient care and the many other responsibilities that physicians have as educators and researchers, are part of professional life. We owe it to our trainees to create opportunities for them to learn these valuable skills.

## Competing interests

The authors declare that they have no competing interests.

## Declarations

Resources and secretariat support for this project was provided by the Royal College.

This article has been published as part of *BMC Medical Education* Volume 14 Supplement 1, 2014: Resident duty hours across borders: an international perspective. The full contents of the supplement are available online at http://www.biomedcentral.com/bmcmededuc/supplements/14/S1. This article was submitted in 2011 and peer reviewed in 2012. Final acceptance for publication as part of this supplement was in 2014.

Publication of this supplement was supported and funded by the Royal College of Physicians and Surgeons of Canada. The funding agency played no role in the design, in the collection, analysis and interpretation of data; in the writing of the manuscripts; and in the decision to submit the manuscript for publication. The articles have been through the journal's standard peer review process for supplements. The Supplement Editors declare that they have no competing interests.
